# Supplementary material for: The dynamic Nexus: gap junctions control protein localization and mobility in distinct and surprising ways
Source: Sci Rep. 2020 Oct 12;10:17011. doi: 10.1038/s41598-020-73892-6 (PMC7550573; doi:10.1038/s41598-020-73892-6)

Supplementary material for manuscript:

Title: The dynamic Nexus: Gap junctions control protein localization and mobility in distinct and surprising ways.

Sean McCutcheon[1*], Randy F. Stout[1,2*], Jr., David C. Spray[1]

[*]these authors contributed equally to these studies
[1]Dominick P. Purpura Department of Neuroscience
Albert Einstein College of Medicine,
1410 Pelham Parkway South, Bronx, NY 10461, USA

[2] The New York Institute of Technology College of Osteopathic Medicine, Department of Biomedical Sciences, 101 Northern Blvd., Old Westbury, NY 11586, USA

Corresponding author: sean.mccutcheon@einsteinmed.org

Supplementary Figure 1

Wild-type Cx43

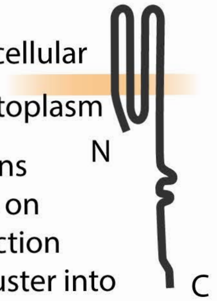

Cx43 connexons form hexameric 4-TMD connexons which doc with connexons on other cells to form gap junction channels Cx43 channels cluster into plaque structures that can be stable or fluid with regard to relative channel arrangement

Cx43 with C-terminus fluorescent protein tag

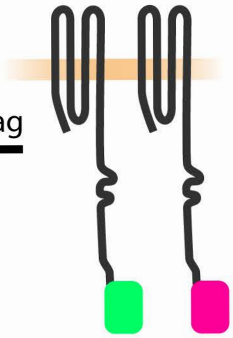

Retains channel function but was shown not to interact with ZO-1

Cx43 with N-terminus fluorescent protein tag

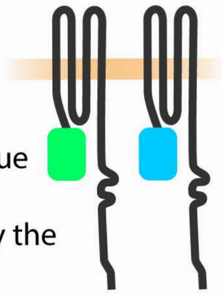

Forms gap juction plaque structures but channel function is abolished by the protein tag

Untagged Cx43 C-terminus 3X cysteine mutant

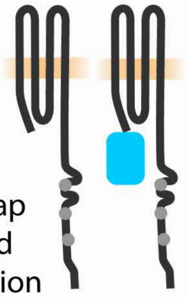

This mutant forms consitutively fluid gap junction plaques and retain channel function and interacts with ZO-1 and occludin

The triple cysteine mutant with an N-terminus fluorescent protein tag forms fluid gap junction plaques but channel function is abolished

Cx43 K258stop truncation

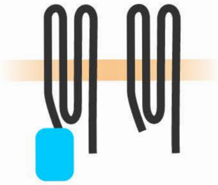

Cx43 truncated at AA 258 or prior forms plaques but does not localize ZO-1 or Occludin to the plaque

Cx30-EGFP

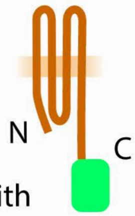

Cx30 forms fluid gap junction plaques and is expressed in astrocytes with Cx43 but does not form heteromeric channels.

CC2-DMPE coumarin linked phospholipid

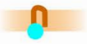

Membrane-tethered mGFP

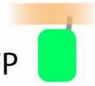

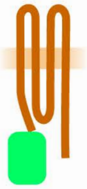

GFP-Cx30
We found that Cx30 intermingles with Cx43 in the gap junction plaque and Cx43 mobility affects Cx3 mobility.

b5Extended-mGFP
1-TMD protein fragment was found to move between stably arranged Cx43 in gap junction plaques

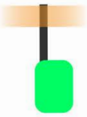

AQP4-mGFP

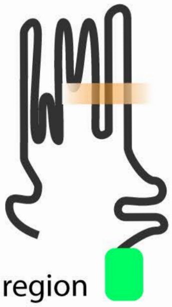

AQP4 is a water channel that can cluster to form square orthogonal arrays of channels that are localized with strong bias to the astrocyte perivascular endfeet where relatively large Cx43 gap junction plaques are also localized. AQP4 is mostly excluded from the membrane region occupied by the Cx43 gap junction plaque structures.

mEmerald-Occludin

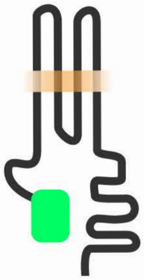

Occludin is a tight junction protein that we find to be localized to Cx43 gap junction plaques only when the C-terminus is intact and not obstructed by a fluorescent protein tag

EAAT2-GFP

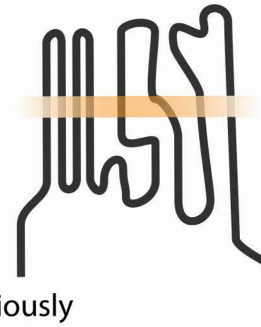

The glutamate transporter (GLT-1 in mice) is excluded from Cx43 gap junction plaques. Localization of this transporter has been shown previously to affect synaptic transmission.

EGFP-ZO1

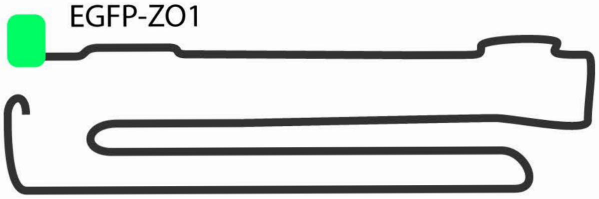

Zona Occludin -1 is considered a tight junction scaffold protein. It is localized to the cell cytoplasm and has been shown to localize to Cx43 gap junction plaques by binding the of the Cx43 C-terminus where it regulates plaque size.

Supplementary Figure 2

Graphical Summary of Results

The Gap Junction Nexus

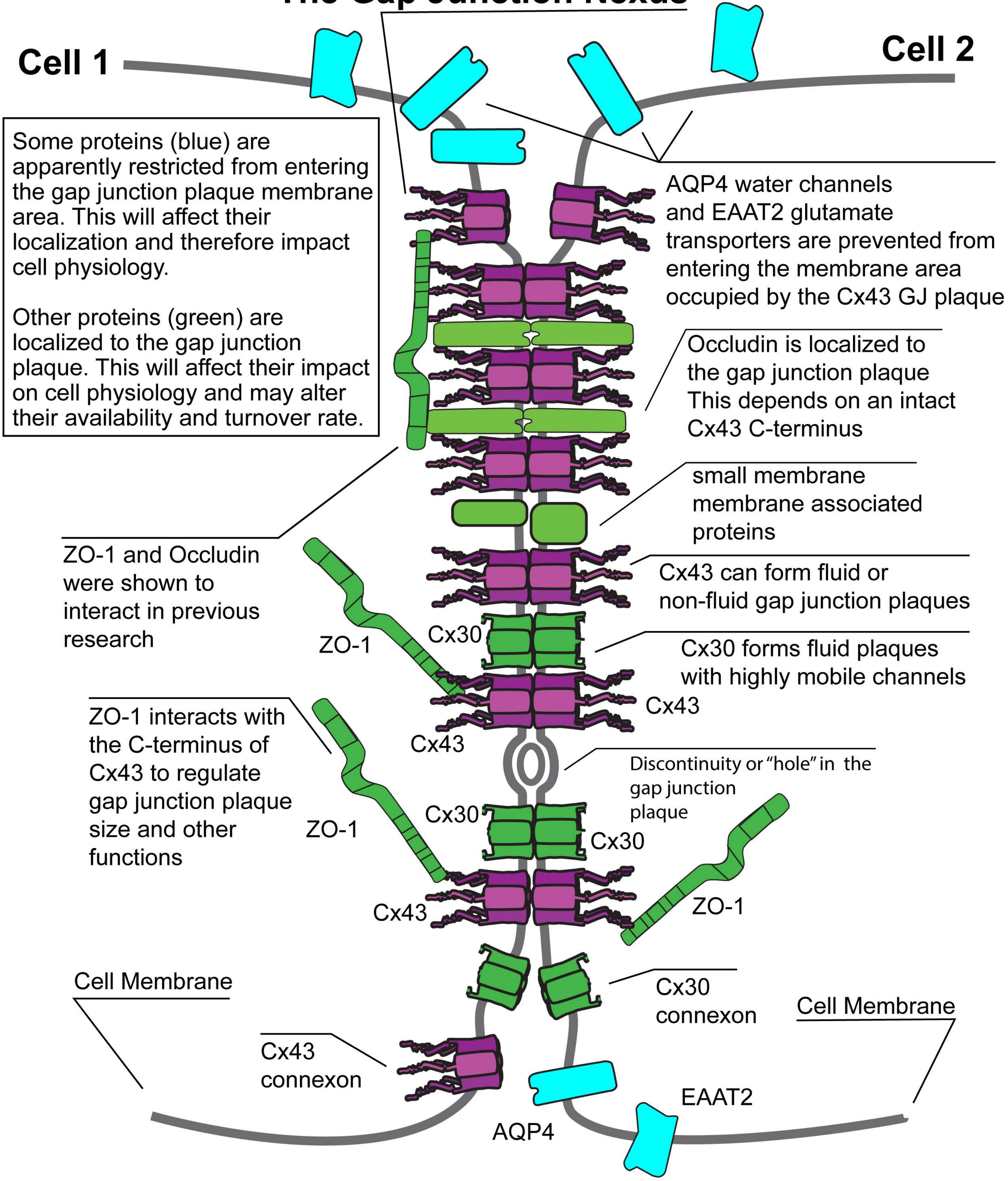

Supplement: Supplementary file 1 — Supplementary Figures. [file 41598_2020_73892_MOESM1_ESM.pdf]
