# Supplementary material for: The dynamic Nexus: gap junctions control protein localization and mobility in distinct and surprising ways
Source: Sci Rep. 2020 Oct 12;10:17011. doi: 10.1038/s41598-020-73892-6 (PMC7550573; doi:10.1038/s41598-020-73892-6)
Supplement: Supplementary file 2 — Supplementary Legends. [file 41598_2020_73892_MOESM2_ESM.pdf]

Supplemental Figure 1. A chart of drawings depicting various connexin and other gap junction nexus components used in this study with comments on the mobility characteristics as described in previous studies and the effects of fluorescent protein tags on channel function and gap junction structure (not drawn to scale and not comprehensive of all scientific findings for the proteins shown). Magenta fusion protein tag (FP) is mCherry. Green FP is EGFP or mEmerald. Blue FP is EBFP.

Supplemental Figure 2. Author's drawing to summarize results of the present study in which we examined the effect of the gap junction plaque structure and fluidity on localization of other proteins that are co-expressed and localize to the same subcellular compartment as Cx43 gap junctions. Gap junction proteins shown in purple. Gap junction plaque permeable proteins/lipids shown in green. Plaque excluded proteins shown in blue.
